# Microplastic Release from Single-Use Plastic Beverage Cups

**DOI:** 10.3390/foods13101564

**Published:** 2024-05-17

**Authors:** Selen Akbulut, Perihan Kubra Akman, Fatih Tornuk, Hasan Yetim

**Affiliations:** 1 Department of Food Technology, Vocational School of Health Services, Uskudar University, 34674 Istanbul, Türkiye; 2 Department of Food Engineering, Faculty of Chemical and Metallurgical Engineering, Yildiz Technical University, 34349 Istanbul, Türkiye; pkcicek@yildiz.edu.tr (P.K.A.); fthtrnk@gmail.com (F.T.); 3 Department of Nutrition and Dietetics, Faculty of Health Sciences, Sivas Cumhuriyet University, 58140 Sivas, Türkiye; 4 Food Engineering Department, Faculty of Engineering and Natural Sciences, Istanbul Sabahattin Zaim University, 34303 Istanbul, Türkiye; hasan.yetim@izu.edu.tr

**Keywords:** microplastics, water, disposable cups, food safety, human exposure

## Abstract

Microplastics (MPs) have attracted considerable attention as one of the most remarkable food and drink pollutants in recent years. Disposable cups, which are widely used as single-use containers, have been suspected as the primary sources of MPs found in cold and hot beverages. In this study, the effect of different exposure times (0, 5, 10 and 20 min) and temperatures (4 °C, 50 °C and 80 °C) on MP release from the single-use cups made of four different materials [polypropylene (PP), polystyrene (PS), polyethylene (PE) coated paper cups and expanded polystyrene (EPS)] into the water was investigated. The number of MPs ranged from 126 p/L to 1420 p/L, while the highest and lowest counts were observed in the PP (50 °C for 20 min) and PE-coated paper cups (4 °C 0 min), respectively. Washing the cups with ultrapure water prior to use reduced the MP release by 52–65%. SEM images demonstrated the abrasion on the surface of the disposable cups as a result of hot water exposure. Intensities of FTIR absorbance levels at some wavelengths were decreased by the water treatment, which could be evidence of surface abrasion. The annual MP exposure of consumers was calculated as 18,720–73,840 by the consumption of hot and cold beverages in disposable cups. In conclusion, as the level and potential toxicity of MP exposure in humans are not yet fully known, this study sheds light on the number of MPs transferred to cold and hot beverages from single-use disposable cups.

## 1. Introduction

Plastics are one of the most convenient materials of modern life, which enables them to be widely used in many fields [1,2]. Plastics are found in many branches of industry, especially in chemistry, materials science and the food chain [3,4,5]. Advantages of plastics include low production costs, lightweight, easy portability, barrier properties and high strength. The food and agriculture sector especially takes advantage of plastic packaging materials [6]. However, beginning in the 20th century, the negative environmental effects of heavy plastic consumption have attracted greater attention in the world as a result of the increasing accumulation of plastic waste in the environment. The fact that most plastics are not biodegradable and remain without decay in nature for centuries impacts all ecosystems [7].

Microplastics (MPs) are defined as plastic particles of less than five millimeters in length (<5 mm), arising from the reduction of waste plastics by the effect of sunlight, wind, flows, living organisms or deliberation by industrial activities [3,4,5]. MPs are low-density and chemically stable synthetic materials and they are classified into two groups, primary and secondary MPs [8,9,10]. Plastic particles in products that are produced at the microscale for industrial and domestic purposes are called ‘primary MPs’, while those formed as a result of the fragmentation of large plastic particles are ‘secondary MPs’ [8,9].

MPs are taken into the body in three main ways: oral intake, inhalation or skin contact [11,12]. Human skin is not likely to allow MPs to pass through, however, individual susceptibility may change due to varying skin pore sizes, meaning that particles smaller than 25 µm can penetrate through the pores [13]. Furthermore, nanoplastics (NPs), and some other toxic additives used in the production of plastics, constitute possible routes of transmission through areas such as open wounds, sweat glands and hair follicles [14,15]. Much research has demonstrated the presence of MP particles in different human organs and structures such as human skin, hair, saliva [15], blood [16], placenta [17,18], meconium [19], feces [20,21] and lungs [22]. MP pollution originating from different sources is encountered in terrestrial ecosystems, especially in aquatic ecosystems, and in the atmosphere. The highest rate of MP pollution is found in aquatic ecosystems, and this pollution rate rises to 60–80% in some seas and 90–95% in some areas [23]. MPs, which are especially common in aquatic ecosystems, have also been detected in the bodies of many living things such as zooplankton, mussels, bivalves, fish, marine mammals and sea birds [24,25].

Food and beverage processing, distribution and pre-consumer post-processing steps are primary sources of MP contamination in food products [26]. The presence of MPs in different types of food and beverages, including beer, honey, soft drinks, cold tea, sugar, eggs, drinking water, mineral waters, salt, tap water, milk and canned fish, has been proven by recent studies [27,28,29,30,31]. Moreover, not only foods, but also the tools used in food preparation and packaging materials have been shown as sources of MP exposure in food and also the human body [32,33,34].

Consumers can be exposed to different amounts of MPs released from plastic packaging materials as a result of use of food preparation tools such as benches [35], blenders [36], microwave heaters, as well as plastic food packaging materials used for the delivery of ready-to-eat foods [5], single-use plastic cups [32,37,38], plastic water bottles, paper coffee cups [39], plastic bottled water and mineral drinks [40,41], hot water drinks prepared with plastic cups and tea bags [42,43].

The factors affecting the number of MPs released from plastic packaging materials and containers into foods are the initial MP concentration of the packaging material, the storage time of the food in the plastic container and the temperature to which the plastic material is exposed to during its production [32,33]. The pH value, hydrophobicity, surface properties and microbiota of a food, and the presence of enzymes, can also affect the transfer of MPs from the contact material to the food matrix [34]. MPs released from food packaging into food have been reported in several recent studies [32,33]. Therefore, the main objective of this study was to determine the effect of using water at different temperatures in disposable plastic cups to determine the presence and characterization of MPs transferred to beverages. In this study, four types of commonly used beverage cups made of polypropylene (PP), polystyrene (PS), polyethylene (PE) coated paper and expanded polystyrene (EPS) were tested to measure the amount of MPs transferred from cups into the water, and the different temperatures (4 °C, 50 °C and 80 °C) and exposure times (0, 5, 10 and 20 min) were selected for analysis by mimicking the general beverage consumption habits with single-use cups. The effect of pre-washing the cups upon MP release into water was also examined.

## 2. Material and Methods

### 2.1. Collection of Plastic Cups

**Figure 1:**
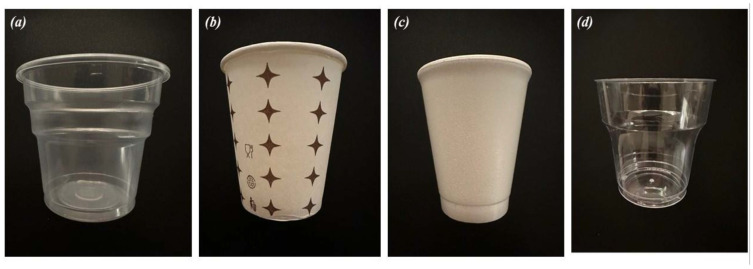
Plastic cups used (**a**) PP cup, (**b**) PE coated paper cup, (**c**) EPS cup (**d**) PS cup.

In the study, four plastic cups, namely PS, PE-coated paper cups, EPS and PP (Figure 1), that are frequently used as single-use beverage containers were purchased from a local retailer in Istanbul, Turkiye between 03/2022 and 04/2023, and the brand, serial number and purchase date of all cups was recorded. The volumes of the selected cups were 200–300 mL. The thickness of the cups was measured using a digital micrometer (Mitutoyo, Kawasaki, Japan). According to the measurement results; EPS: 1880 µm ± 0.005, PP and PE: 350 µm ± 0.01, PE-coated paper cup (PE thickness): 200 µm ± 0.01, PS: 850 µm ± 0.01.

### 2.2. Prevention of Environmental MP Contamination

To prevent MP contamination of the samples, only glass materials were used throughout the study. All glass materials were rinsed three times with ultrapure water before being used in the analysis. Experiments were performed in a HEPA-filtered chamber (Thermo Scientific, S2020 1.2, Waltham, MA, USA) using a cotton lab coat and nitrile gloves. Before starting the study, the cabin was cleaned with 70% ethyl alcohol. As a control sample, ultrapure water was kept in a glass beaker, but no contamination was detected. The filters used for filtration were individually packaged sterile filters (Filter Bio, Nantong, China). After filtration, the filters were taken in glass petri dishes cleaned with ethyl alcohol (70%) for further analysis.

### 2.3. Experimental Design and Extraction of MPs from Plastic Cups

**Figure 2:**
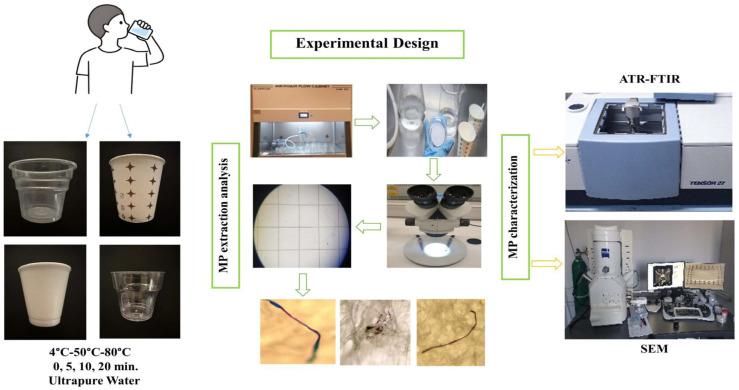
Experimental procedure applied to test MPs release.

In this study, real-use conditions of the disposable plastic cups were mimicked by applying water at different temperatures (4 °C, 50 °C and 80 °C) [44] and exposed for different times (0, 5, 10 and 20 min) [32,33]. The experimental procedure is schematized in Figure 2. Firstly, all plastic containers were rinsed with ultrapure water at room temperature (~25 °C) and dried at ambient temperature prior to the experiment. Unrinsed cups were also tested for the determination of the pre-rinsing effect. Ultrapure water (50 mL) at 4 °C, 50 °C or 80 °C was dispensed to the cups and held for 0, 5, 10 and 20 min at room temperature. In total, 50 mL of ultrapure water filled 20–27% of the plastic cups. At each time interval, water samples in an individual cup were removed for MP sampling. Standard filtration techniques were applied to determine the amount of MPs released from the plastic cups. All the samples were filtered through a sterile nitrocellulose membrane filter (0.45 µm pore size, Filter Bio, China) using a vacuum filtration set (Isolab, Eschau, Germany). In the preliminary studies, the filter was stained with Rose Bengal (200 mg/L) in order to determine the possible contamination of natural organic particles [27]. After leaving the filters for 5 min for reaction, they were washed with ultrapure water and the stain was drained. Non-stained particles were referred to as MPs during the microscopic evaluation because Rose Bengal, a biological origin stain, could bind to only non-synthetic materials [45]. After filtration, the filters were stored in pre-cleaned glass petri dishes at room temperature for further analysis. All the experiments were performed in triplicate. 

### 2.4. MP Quantification and Characterization

After filtration, the filters were examined under a dissecting microscope (Isolab, Germany) at 10× magnification, and the observed MPs were counted and recorded by visual evaluation. The color and shape characteristics of the observed plastic particles were also determined. A total of 156 samples were counted at room temperature (25 °C) [45]. In the study, the particles on the filter were examined under a camera light microscope (Olympus, CKX41, Tokyo, Japan) with a 20× objective, and their sizes were measured. The particles with measured sizes were categorized into four classes (<10 µm, 10–50 µm, 50–100 µm and >100 µm) [38].

### 2.5. SEM

SEM analysis was performed to observe the morphological changes on the inner surface of the cups caused by the use of water at different temperatures. For this purpose, unused plastic cups were compared with plastic cups treated with ultrapure water at 4 °C, 50 °C or 80 °C for 20 min. For the SEM analysis, 1 cm × 1 cm sections were prepared from the samples and analyzed for a total of 16 samples. The plastic surface was coated with gold palladium for 18 mA 150 s with a coating device (Quorum, SC7620, Nottingham, UK). Then, imaging was performed using a Scanning Electron Microscope (SEM, Zeiss, EVO MA10, Oberkochen, Germany) at 10 KV accelerating voltage in the magnification power range of 50–3000× [5,38].

### 2.6. ATR-FTIR

Plastic cups exposed to water at different temperatures were used in order to investigate the chemical properties of the surface of plastic cups. Unexposed plastic cups were compared with plastic cups treated with ultrapure water at 4 °C, 50 °C or 80 °C for 20 min. After exposure, the cups were cut into 1 cm × 1 cm square pieces and measurements were taken using the ATR-FTIR spectrometer (Bruker Tensor 27, Bremen, Germany). Three pieces from each cup were analyzed using different points. Spectra were recorded in the 4000–600 cm^−1^ range with a total of 36 scans [38,46].

### 2.7. Estimation of MP Exposure by Using Disposable Plastic Cups

The average daily consumption of hot or cold beverages using disposable plastics in Turkiye was assumed to be around 0.5–1.0 L [47]. By adapting the data we obtained from our study to consumer habits, we calculated how much MPs consumers would be exposed to: maximum MP production × L × 52 weeks [5].

### 2.8. Statistical Analysis

Statistical analysis was performed using a Windows-based statistical analysis program (IMB SPSS 24, Richmond, VA, USA). Two-way analysis of variance (ANOVA) was performed, and the statistical differences between the means were evaluated at the significance level of 95% by using the Tukey test. The effect of washing applied to the cups was analyzed with a paired sample t-test. All analyses were performed in triplicate.

## 3. Results and Discussion

### 3.1. Characteristics of Released MPs from Plastic Cups into Beverages

In this research, MPs released from the most commonly used plastic cups were determined at different exposure temperatures, and these temperature parameters were selected with reference to the daily consumption habits of the consumers. The time periods were based on the information stated in a survey that consumers prefer a drink to consume within an average of 15 min [39]. Thus, how many MPs are exposed by the consumers during the period of hot or cold beverage consumption using single-use plastic cups was analyzed. The main material type of the cups, made of four different plastic materials and obtained from places easily accessible for consumers, was matched directly to the material using FTIR, similar to previous studies [5,28,32,38].

**Figure 3:**
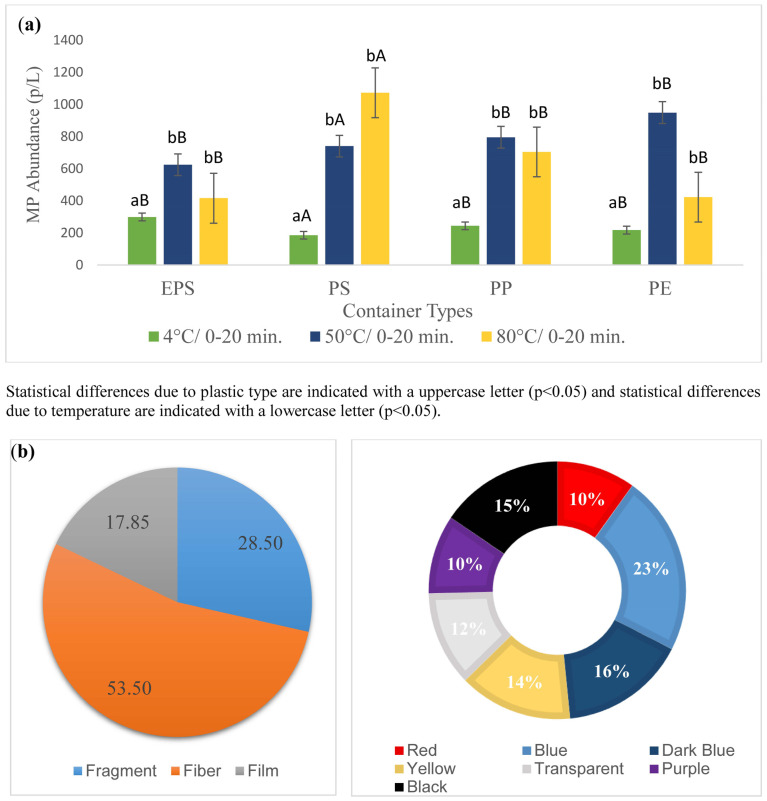

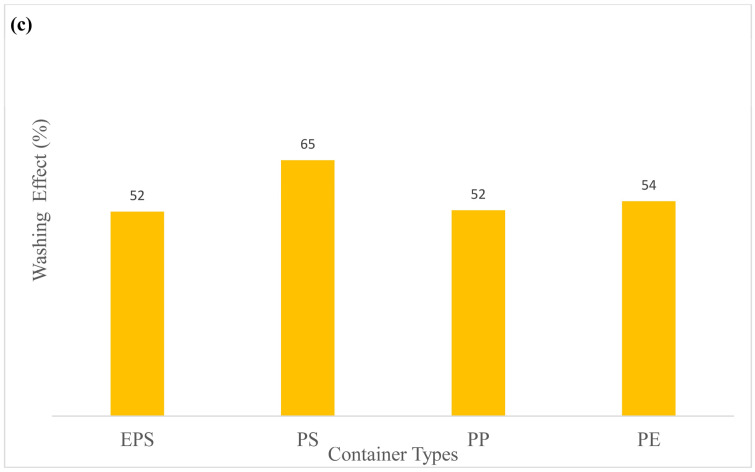
Microplastic released by four types of plastic cups at different temperatures and times (**a**), Mean quantity of MPs exposed during the consumption period and MP color and shape (**b**), Decrease in the number of MP particles of pre-washing (**c**). PP: Polypropylene, PS: Polystyrene, PE: Polyethylene coated paper cups, EPS: Expanded polystyrene.

The effect of the different temperatures and exposure times on the MPs transferred into the water was analyzed, and the results are shown in Figure 3. The average MP transfer observed in all experiments was 556.80 ± 31.39 particles/L (p/L). The lowest and highest MP numbers were 126.6 ± 75.71 p/L and 1420 ± 380 p/L at 4 °C for 0 min in PE-coated paper cups and 50 °C for 20 min in PP cups, respectively. Figure 3 shows the average MP release depending on temperature and time, mean quantity of MPs exposed during the consumption period, MP color and shape and decrease in the number of MP particles during washing, respectively. In terms of the type of plastic, the minimum and maximum MP values were 153 ± 30.55 − 1360 ± 363.8 p/L in PS cups, 160 ± 20 − 1420 ± 380 p/L in PP cups, 126 ± 75.71 − 1346 ± 473.84 p/L in PE-coated paper cups and 246 ± 100.66 − 720 ± 393.95 p/L in EPS cups, respectively. All the analyses showed that the use of plastic cups containing water at different temperatures had a statistically significant effect on the release of MPs. As seen in the figure, MP release increased with the increasing temperature. MPs released at 50 °C and 80 °C were significantly (*p* < 0.05) higher than those of 4 °C. Considering 50 °C and 80 °C, the number of particles was not significantly (*p* > 0.05) different. The effect of exposure time on MP release was not also statistically significant (*p* > 0.05). The release of MPs from PS cups was found to be statistically significant compared to PP, PE and EPS cups (*p* < 0.05). In previous studies examining MPs released from different plastic food packaging materials, a higher MP release was found in PS packaging than in HDPE bags, PP takeaway cups and PP, PE and PET containers [5,32]. This supports the idea that PS cups could pose a high MP risk by hot and cold beverage consumption compared to other plastic cups. The number of MPs transferred to the water with the increasing temperature revealed were generally higher independently from the type of plastic cups (Figure 3a). In one study, in which 5 °C and 60 °C temperature application was investigated in three different plastics types, as similar to our findings, water temperature at 60 °C caused a higher MP release in the PP and PE-coated cups compared to at 5 °C [38]. This could be due to the increase in the deformation rate on the surface of the plastic material with the increasing water temperature, which increases the tendency of MP release.

In color characteristics, the most dominant color was blue (23%), while dark blue (16%), black (15%), yellow (14%), transparent (12%), red (10%) and purple (9.9%) were also detected. In this study, the shapes of MP particles were determined as 53.5% in fiber, 28.5% in fragment and 17.8% in film form. The color and shape findings of MPs detected in this study were in accordance with the results in several food samples as reported by several researchers [31,32,41]. In studies examining the presence of MP in soft drinks packed in different materials (PET bottle, Tetra Pak packaging) and honey samples, the most common form of MP was fiber, while MP particles in film and fragment forms were also observed [31,48]. 

The size of the particles measured was 11.11% <10 µm, 53.55% 10–50 µm, 13.33% 50–100 µm and 24.44% >100 µm. In the size distribution, the most dominant MP size was found to be in the size range of 10–50 µm. In a study examining the release of MPs from plastic cups, the vast majority of samples were <50 mm in size [38], and in another study, 55% of the detected particle size was <500 μm [32].

### 3.2. Effect of Pre-Washing on MP Release

In this study, it was found that the pre-washing treatment reduced the MP number by 52–65% (Figure 3c). Therefore, it could be speculated that washing the disposable plastic cups with water before use could reduce the level of MPs transferred into the beverage. The average MP values after pre-washing the PS, PP, EPS and PE-coated paper cups exposed to water at different temperatures and times were 667.22 p/L, 582.22 p/L, 530.55 p/L and 447.22 p/L, respectively. Similarly, Chen et al. [49] found varying levels of MPs released in different types of plastic cups in water. This variation might be due to several reasons such as different water contact areas and water levels in the cups, varying plastic types and their properties originating from different processing conditions [32,49]. 

Similar results to our findings were reported by Zhou, Wu, Tang, Chen, Cheng, Wei, Ma and Liu [38] who investigated the level of MPs released from different plastic cups (PET, PE and PS) and the effect of pre-washing the inner surface of the packaging before use. They found that the number of MPs was reduced by 35.9-50.2% by pre-washing. According to another study, it was reported that washing and repeated use of disposable plastic food packaging increased MP release and re-washing (100×) take-away containers was specified as the main factor that increased the number of MPs released [5]. The presence of MPs in the cups before food exposure may originate from the cup manufacturing processes, such as extrusion and paper coating. Although studies often focus on the release of MPs, chemicals (e.g., some toxic chemicals), heavy metals (cadmium, arsenic, etc.), stabilizers, colorants, pharmaceuticals and persistent organic pollutants (POP) and even pathogenic microorganisms could also be transferred to the liquid foods from the plastics [1,33,35,50,51].

### 3.3. SEM 

**Figure 4:**
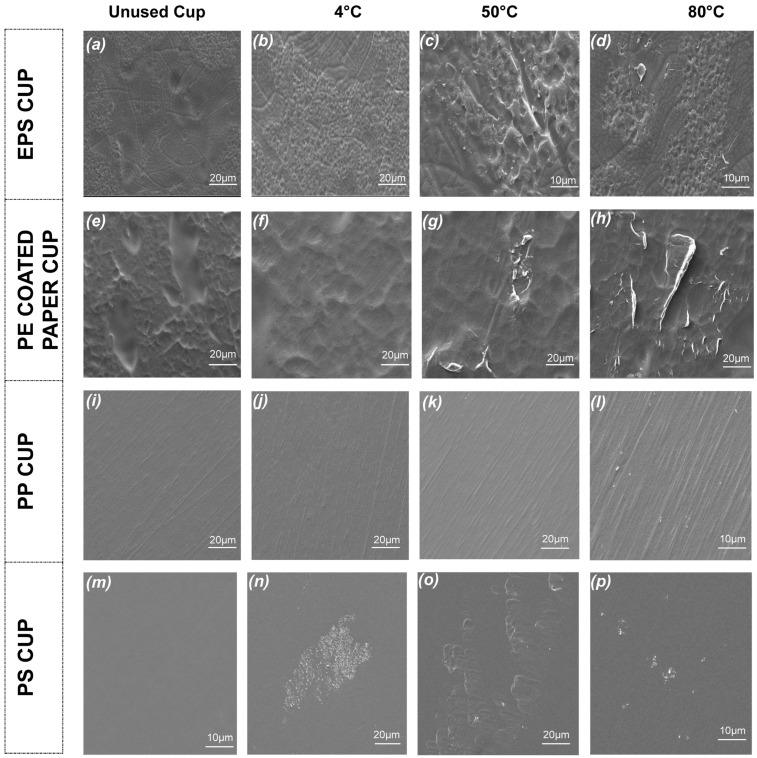
The effect of hot water application on the inner surfaces of plastic cups under scanning electron microscope (1000×–3000×). PP: Polypropylene, PS: Polystyrene, PE: Polyethylene coated paper, EPS: Expanded polystyrene.

The morphological change in the inner surfaces of the cups was observed using SEM analysis (Figure 4), and untreated cup surfaces and 20 min exposures (at 4 °C, 50 °C and 80 °C) counterparts were compared. As can be seen from the SEM images, the surfaces of the plastic cups exposed to water caused noticeable deformations depending on the temperature. Higher levels of abrasion on the surfaces were observed as the water temperature elevated, which likely caused higher numbers of MP breakage from the surfaces. The surface deformation on the inner surface of plastic cups can be one of the main causes of MP release into food [32,46,49]. 

According to the SEM results, unused PP (Figure 4i–l) and PS (Figure 4m–p) cups had smoother, more uniform and dense surfaces. With the increasing temperature, porous structures and deformation were observed, especially on the surface of PS and PP cups which were smoother before use. The higher MP release from the PS and PP cups at 80 °C also supports the literature findings. Similarly, Chen et al. [49] reported that the subjection of the PP and PS cups to water at 95 °C for 20 min caused more deformation on the inner surface of the cups than subjection temperatures of 10 °C, 40 °C or 70 °C.

PE-coated paper cups (Figure 4e–h) are produced by coating PE film on the inner surface of paper cups, which results in high surface roughness and bumps even on unused cups [32,38]. In addition, the thickness of the PE coating on the paper cups is about 50 µm, which is much thinner than those of PP and PS cups [52]. This can also cause the release of MPs from the cup surface much more easily by the effect of environmental conditions such as high temperature and the acidity of food. In this study, due to the increasing water temperature, PE-coated paper cups were exposed to serious abrasion. Thus, the breakage and fractures on the surface likely caused MP release. In the case of the PE-coated cups, where the highest MP transition was detected as a result of the water exposure at 50 °C, tears and ruptures on the film surface were also visually noticeable. Similar findings were obtained in some studies conducted on paper cups in which high surface corrosion occurred due to the higher water temperatures [38,39]. In EPS cups (Figure 4a–d), similar to PE-coated cups, bumps were observed in the unused cups due to the foam structure, and the increase in water temperature resulted in abrasions on the inner surface of the cups, which was one of the main causes of MP ruptures.

### 3.4. ATR-FTIR

**Figure 5:**
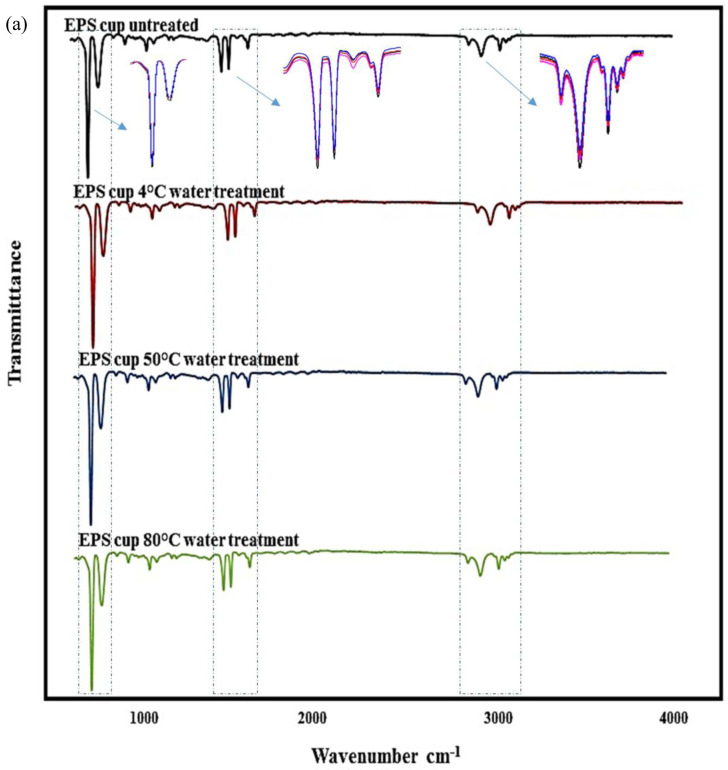

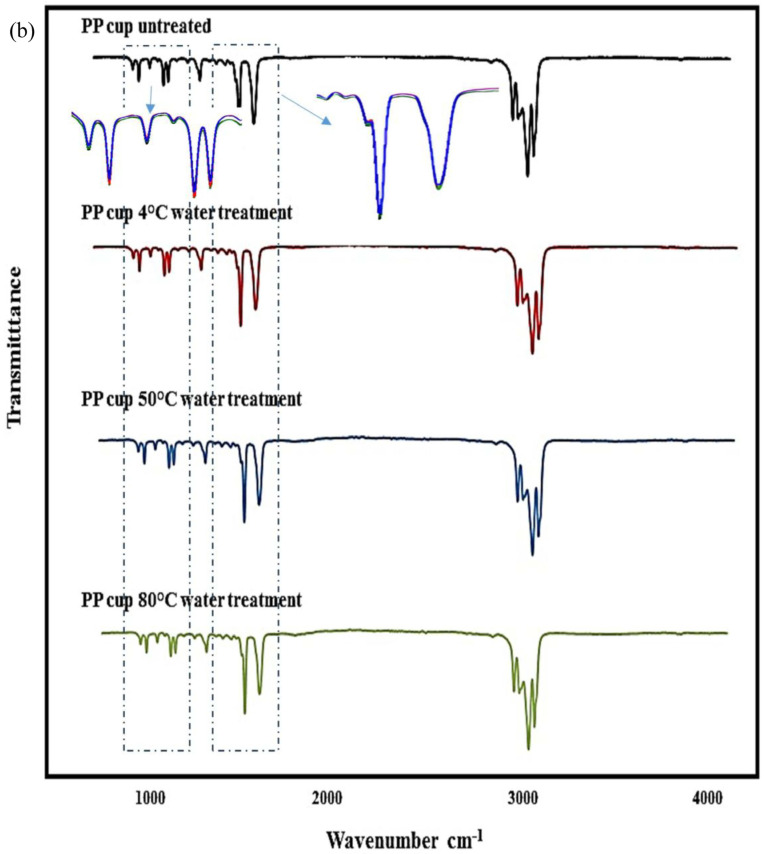

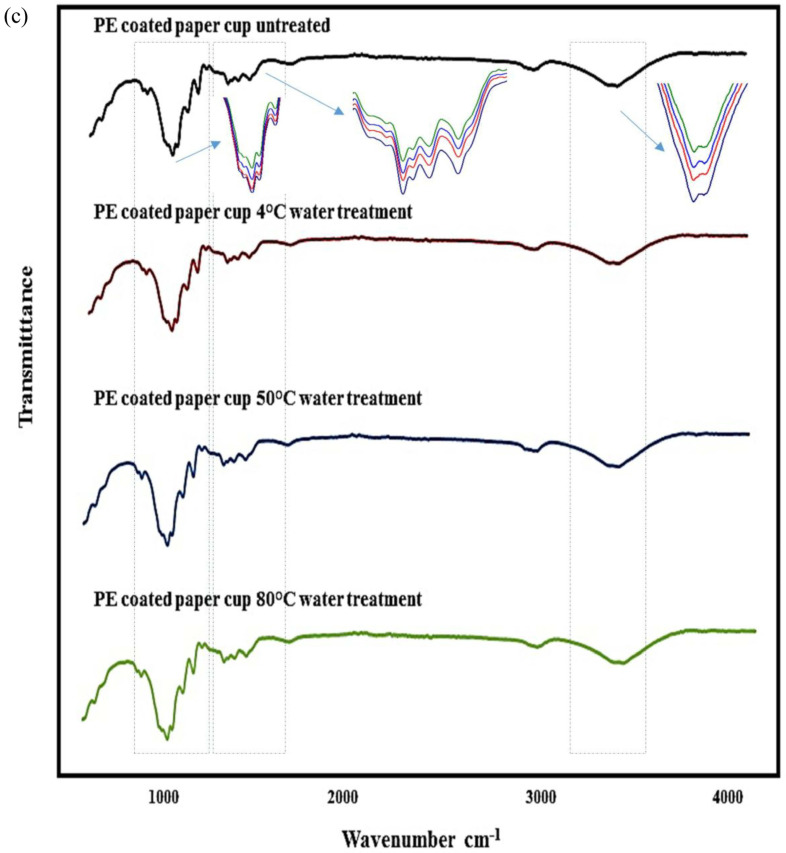

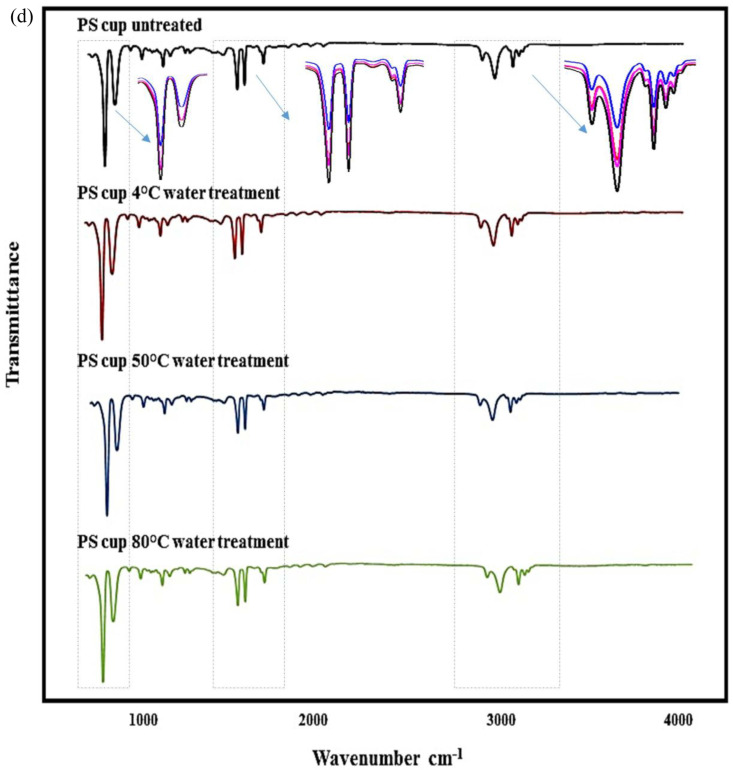
The FTIR spectra before and after water exposure of plastics at different temperatures. (**a**) EPS: Expanded polystyrene cup, (**b**) PP: Polypropylene cup, (**c**) PE: Polyethylene coated paper cup, (**d**) PS: Polystyrene cup.

The water-treated and untreated cups were analyzed using ATR-FTIR to characterize the molecular structure of their surface. In Figure 5, the reference peaks for the respective plastic cups were compared, and their characteristic peaks are shown on the spectra. The type of cups was verified using FTIR analysis concluding high similarity (<99.9%) with the respective plastics present in the OPUS spectral library. As seen in the figure, plastic cups exhibited similar spectra as a result of water exposure at different temperatures. However, the intensities of the peaks at some wavelengths decreased with the increasing temperature, which demonstrates the deformation of the plastic resin constituents. Abrasion of the inner surfaces of the plastic containers using heat treatment (surface erosion) may cause a decrease in IR beam intensity. The observed change in absorption peaks can be attributed to the possible degradation of chemical bonds on the plastic surface, while electrochemical factors and the applied temperatures can be associated with changes in the layer structure of the polymer surface [37,53].

In the case of the PP cups, peaks belonging to C-H stretching were observed in the 2950–2838 cm^−1^ region, while CH_2_ vibrations were observed at the wavelength of 1455 cm^−1^. In addition, 1377 cm^−1^ represented CH_3_ bands, and all reference peaks of the polymer (C-CH_3_ band, C-C band, C-C stretch) were observed in the range of 808–1166 cm^−1^ region. In the case of the PS and EPS cups, all the peaks, called fingerprints, of the polymers were observed. These characteristic peaks are a 3024 cm^−1^ region, aromatic C-H stretches, 1492–1601 cm^−1^ aromatic ring structure stretches, 1027 cm^−1^ aromatic CH band, the 756 cm^−1^ peak is the CH band and 694 cm^−1^ is the largest peak of the ring structure other than the aromatic CH band [54]. When the FTIR spectrum of the PE-coated paper cup is examined, asymmetric/symmetric CH_2_ stretching was observed in the range of 2916–2850 cm^−1^, while 3334 cm^−1^ and 1426 cm^−1^ CH_2_ bands were the characteristic peaks belonging to the cellulose on the outer part of the paper cup [46,55]. The observation of all the peaks mentioned in the cups at all temperature levels was the expression that there was no chemical change on the inner surface of the cup. Similarly, Zhou, Wu, Tang, Chen, Cheng, Wei, Ma and Liu [38] reported that different temperature applications did not cause any chemical changes on the inner surface of the PP, PET and PE plastic cups. On the other hand, the characterization of MPs by FTIR is not completely possible due to releasing other additives such as colorants, chemicals and fluorescent compounds, altering the polymer spectra and, therefore, preventing comparisons with the chemical library databases [5].

### 3.5. Human Exposure to MPs Originating from Plastic Cups

The culture of eating outside, which has become a part of the daily life of modern societies, has increased quietly in recent years. The catering sector and catering apps’ rapid development have made it easy for consumers to order food from anywhere [32]. Most of the ready-to-eat food and beverages consumed outside are served in disposable cups and boxes made of plastic materials, which increases the use of plastic food packaging used directly in ready-to-eat food services [35,46,48]. The most common use of disposable plastic cups is during the consumption of hot and cold drinks. Disposable plastic cups are used extensively for the consumption of beverages of different qualities and contents, ranging everywhere from public transport (e.g., intercity bus services, airplanes, etc.), to cafeterias, school canteens and homes. For example, according to statistics, 50 billion plastic cups are used annually in the USA alone [56]. As presented earlier (Figure 3a), the highest maximum MP was 720–1420 p/L. The annual MP exposure of an adult by consuming beverages by PS PP, PE-coated paper and EPS cups were 35,360–70,720 MP, 36,920–73,840 MP, 35,013–70,026 MP and 18,720–37,440 MP particles, respectively. Cox et al. [57] found that consumers are exposed to MPs in the range of 39,000–52,000 per year through food and beverages. When inhalation MPs are added to this data, this figure increases up to 74,000–121,000 particles per year, per person.

## 4. Conclusions

In this study, it was determined how many MPs were released into the water, with simulated cold and/or hot beverage consumption conditions using single-use plastic cups at different temperatures (4 °C, 50 °C or 80 °C) with the consumption periods (0, 5, 10 or 20 min). Increasing the water temperature and extending the exposure time caused higher releases of MPs into the water. Pre-washing plastic cups with water before use was an effective way to reduce MP counts by 52–65%. The FTIR results showed that no molecular change was obtained on the inner cup surface by water exposure, while the reduction in the intensities of the peaks in some specific wavelengths was observed with the increasing water temperature. SEM images demonstrated the deformation of the plastic cup’s surfaces by the treatment, which likely resulted in an increase in MP release. An MP risk assessment showed that the annual MP intake of an individual who consumes hot or cold beverages with disposable plastic containers once or twice a week was estimated to reach a number of 18,720–73,840 particles. In conclusion, this study suggested that single-use plastic beverage cups were effective sources of MP release, which means that the use of disposable plastic cups should be minimized by substituting their alternatives.

## Data Availability

The original contributions presented in the study are included in the article, further inquiries can be directed to the corresponding author.
